# Real-world occupational therapy interventions for early-stage dementia: Characteristics and contextual barriers

**DOI:** 10.1177/14713012241272815

**Published:** 2024-08-20

**Authors:** Bethan M Edwards, Monica Busse, Teena J Clouston, Ben Hannigan

**Affiliations:** Service User Research Enterprise (SURE), Institute of Psychiatry, Psychology and Neuroscience, 34426 King’s College London, UK School of Healthcare Sciences, 2112 Cardiff University, UK Research and Development Department, Cwm Taf Morgannwg University Health Board, UK; Centre for Trials Research, 2112 Cardiff University, UK; School of Healthcare Sciences, 2112 Cardiff University, UK

**Keywords:** dementia, occupational therapy, rehabilitation, everyday functioning, activities of daily living

## Abstract

**Aim:**

There is an absence of evidence generated in a UK context to support interventions based on occupational therapists’ core skills for people living with early-stage dementia. To inform the development of a programme theory and a future evaluation, this paper aimed to describe real-world (routine) community-based occupational therapy interventions for this population and contextual barriers.

**Method:**

Occupational therapy practitioners (*n* = 21) from five Health Boards in Wales, UK participated in semi-structured interviews (*n* = 17) which were audio recorded, transcribed, and analysed thematically.

**Findings:**

The availability of, and access to, real-world community-based interventions was variable, and associated with multilevel contextual barriers (resources, understanding of dementia specialist occupational therapy, professional influence, and evidence base). Where available and accessible, contents comprised a pre-intervention component (relational work, assessment, and goal setting) and intervention component (personalised problem-solving and coping strategies, emotional support, and advice and signposting), to meet needs associated with everyday activities and poor wellbeing. Variation in mode, duration, contents, and who received interventions, was associated with contextual barriers.

**Conclusion:**

Findings indicate that the development of an intervention programme theory and future evaluation design, will need to account for the impact context may have on the variability of real-world intervention characteristics, and how this in turn may influence outcomes.

## Introduction

Globally, 57.4 million people are estimated to be living with dementia, with prevalence expected to increase to 152.8 million by 2050 (GBD 2019 Dementia Forecasting Collaborators, 2022). In addition to a chronic and progressive decline in cognitive functioning, a significant deterioration in ability to perform everyday activities independently is required for a dementia diagnosis (World Health Organisation, 2021). Dementia is consequently a leading cause of disability globally (GBD 2016 Neurology Collaborators, 2019), with declining wellbeing and quality of life associated with diminishing independence (Giebel et al., 2014, 2015). In the absence of a cure, providing support to enable people to live well with dementia is therefore a central tenet of policy worldwide (Alzheimer Europe, 2014; Quinn et al., 2022). This includes initiatives supporting early diagnosis and interventions, including psychosocial interventions that aim to maintain independence and participation in everyday activities (Alzheimer Europe, 2014; Royal College of Psychiatrists, 2018; Welsh Government, 2018; World Health Organisation, 2017). In the UK, the National Institute for Health and Care Excellence (NICE) (2018) recommends clinicians consider occupational therapy for people living with early-stage dementia to achieve this aim.

Despite national recommendations, there is an absence of evidence generated in a UK context demonstrating the effectiveness or efficacy of community-based occupational therapy interventions for people living with early-stage dementia (Edwards, 2022). Only one UK RCT has been reported, which identified no differences between the experimental intervention, Community Occupational Therapy in Dementia (COTiD), and treatment as usual (TAU) (which included routine, or real-world occupational therapy interventions at some sites) (Wenborn et al., 2021). Inconsistent outcomes have also been reported internationally in relation to COTiD, with the impact of differing contexts (Voigt-Radloff et al., 2011; Wenborn et al., 2021), contextual implementation barriers (Voigt-Radloff et al., 2011; Walton et al., 2019; Wenborn et al., 2021) and the confounding impact of comparison or control groups consisting of varying types of occupational therapy interventions (Voigt-Radloff et al., 2011; Wenborn et al., 2021), identified as potential contributory factors. Alternative community-based occupational therapy interventions for people living with early-stage dementia have been evaluated, albeit less extensively, by Ávila et al. (2018), Coe et al. (2019), and Dooley and Hinojosa (2004); however, they have significant methodological weaknesses (e.g., have not used a RCT design, or were small pilot studies) and demonstrate considerable heterogeneity in intervention characteristics (e.g., mode, duration, intensity, contents and components).

Inconsistent outcomes reported by RCTs evaluating COTiD, and the heterogeneity of other occupational therapy intervention programmes, raise significant uncertainties about the optimum characteristics of occupational therapy interventions for this population; in addition to the impact context has on these characteristics and associated outcomes. Reflecting these uncertainties, this paper forms part of a larger piece of work seeking to develop an intervention programme theory to inform a future evaluation of real-world occupational therapy interventions for people living with early-stage dementia in the community. The development of a programme theory (theory of change), is recommended by the Medical Research Council's (MRC) framework for complex intervention research when developing and evaluating complex interventions (Skivington et al., 2021). Programme theories typically describe the characteristics of an intervention, how it leads to specific outcomes (its mechanisms), and how context interacts with the intervention (O’Cathain et al., 2019). Unfortunately, programme theories have been absent from existing occupational therapy interventions for this population, resulting in the need to develop one in order to investigate the uncertainties outlined (Edwards, 2022). This paper reports on work contributing to this broader aim, by exploring the characteristics (e.g., mode, location, duration, intensity, contents and components) of real-world occupational therapy interventions for people living with early-stage dementia in the community, and contextual barriers.

### Real-world interventions: Existing evidence

Research about the characteristics of real-world community-based occupational therapy interventions for people living with early-stage dementia is scarce. Existing evidence derives from brief surveys of practice generating quantitative data (Bennett et al., 2011; McGrath & O’Callaghan, 2014; Stigen et al., 2023; Swinson et al., 2016), audits (Abendstern et al., 2017), and qualitative studies using small samples (Cummins & Warren, 2010; Kinsella et al., 2023). Aside from Cummins and Warren (2010), these studies have not had a specific focus on early-stage dementia and report data generated about practice with people living with dementia of all stages together (Bennett et al., 2011; McGrath & O’Callaghan, 2014; Swinson et al., 2016); people living with cognitive impairments (including dementia without specification of stage) (Stigen et al., 2023); and older adults with mental health needs (including dementia without specification of stage) (Abendstern et al., 2017). Another limitation consists of the absence of a specific focus on community-based interventions by Bennett et al. (2011) and McGrath and O’Callaghan (2014), who report on data pertaining to multiple settings (e.g., community, hospital, care home) together.

With these caveats, two UK based studies suggest that the availability of real-world interventions is variable (Swinson et al., 2016), that intervention contents commonly include environmental adaptations, assistive technology and equipment (Abendstern et al., 2017; Swinson et al., 2016), that input is brief (median duration of 2.5 hours) (Swinson et al., 2016), and that non-profession specific activities (e.g., care co-ordination), are undertaken (Abendstern et al., 2017; Swinson et al., 2016). International surveys of practice in Ireland (McGrath & O’Callaghan, 2014), Australia (Bennett et al., 2011), and Norway (Stigen et al., 2023) have also identified environmental adaptations, equipment, and assistive technology as the most commonly reported intervention content.

Contextual barriers have not typically been the primary focus of existing research, and knowledge is limited in this area. In the UK, Swinson et al. (2016) reported barriers prescribing equipment and/or assistive technology, whilst Kinsella et al. (2023) described challenges at an organisational level (risk cultures) and at a team level (maintaining team relationships), which restricted the implementation of research evidence into routine practice. Internationally, organisational barriers associated with a lack of financial resources (Cummins & Warren, 2010), a lack of time (Bennett et al., 2011; McGrath & O’Callaghan, 2014), long waiting lists (Cummins & Warren, 2010), organisationally imposed role restrictions (Bennett et al., 2011; McGrath & O’Callaghan, 2014), disjointed services (Cummins & Warren, 2010) and a lack of therapist knowledge and skills (Bennett et al., 2011; McGrath & O’Callaghan, 2014) have been identified.

### Aim

This paper reports on work contributing to the development of an occupational therapy intervention programme theory. Objectives consisted of describing: 1. The characteristics (e.g., mode, location, duration, intensity, contents and components) of real-world community-based occupational therapy interventions for people affected by early-stage dementia; and 2. Contextual barriers to the implementation of these.

## Materials and method

### Sampling and recruitment strategy

Occupational therapy practitioners (occupational therapists and occupational therapy assistants or technicians) were recruited using a purposive sampling strategy from five NHS Health Boards between 21.3.2018 – 1.11.2018 in Wales, UK. In one Health Board, practitioners were invited to participate by the first author (BE) using an invitation email. Occupational therapy managers acting as gatekeepers approached practitioners via invitation email in the remaining four Health Boards. Inclusion criteria comprised: - 1. Working as an occupational therapy practitioner in dementia specialist mental health services; and 2. Willing to participate and able to provide informed consent.

### Data generation

Data were generated utilising semi-structured interviews to enable a detailed understanding about:- 1. The impact early-stage dementia has on everyday activities; 2. Interventions delivered by occupational therapy practitioners working with people living with early-stage dementia; and 3. The intervention implementation context (including the identification of barriers). An interview schedule was developed, with interview questions pertinent to this paper seeking to explore intervention rationale (e.g., aims, underpinning research and theory), intervention characteristics (e.g., content, duration and intensity, mode) and intervention delivery or implementation barriers as defined by participants themselves. Interviews occurred on one occasion at practitioners’ workplaces and were audio recorded. The first author (BE) conducted all interviews and at one site had an established relationship with practitioners. Interviews were carried out on an individual and group basis in accordance with participant preference.

### Data processing and analysis

In preparation for analysis, interviews were transcribed by an external contractor specialising in the transcription of health-related interviews. Following anonymisation, transcripts were uploaded to NVivo, which was used to manage and store data.

Data were analysed thematically using a pragmatic and systematic approach as advocated by Miles (2014). Firstly, this consisted of the first author (BE), categorising the entire data set in accordance with the three areas covered by semi-structured interviews (described above), with this paper reporting on the analysis of data relating to interventions delivered by occupational therapy practitioners working with people living with early-stage dementia and the implementation context (including the identification of barriers). Findings pertaining to the impact early-stage dementia has on everyday activities is reported elsewhere (Edwards et al., 2024). Secondly, familiarisation by reading and re-reading data occurred, with initial and provisional coding memos made using an a priori coding framework for data pertaining to interventions (Table 1), with initial coding memos developed inductively for contextual barriers. Thirdly, data was read and re-read, and taking an inductive approach, provisional coding memos were revised iteratively, with sub-codes developed as necessary, to ensure codes were grounded in the data (Miles, 2014). Final codes and sub-codes accompanied by data extracts were reviewed by authors (TC, BH and MB) to enhance credibility, with refinements made where required. Fourthly, a narrative description of ‘themes’ (codes) and ‘sub-themes’ (sub-codes) was produced and is presented in this paper.

**Table 1.** A priori coding framework.

A priori coding framework
Rationale
Content and Components
Mode and Location
Interventionist
Duration and Intensity

### Ethics

Consent was obtained from all participants in writing and only participants who were able to provide informed consent were recruited. A Health Research Authority (HRA) Research Ethics Committee (REC) reviewed and approved this study (REC Reference: 18/WA/0107). Institutional approval was received from all five participating Health Boards. All identifying participant and recruitment site details have been anonymised.

### Rigour

Methods to enhance trustworthiness were utilised, including the triangulation of data from multiple sources (21 practitioners) and contexts (five Health Boards) (Lewis et al., 2014). Direct quotations have been used to enable the reader to evaluate whether themes reported are supported by the data, with contradictory perspectives highlighted. As described, at one site a prior established relationship existed between the interviewer (BE) and participants. BE had no managerial or supervisory responsibility for any participants and her seniority was equal to most participants at this site, minimising the impact such responsibilities and seniority could have on participants’ responses. Further strategies were developed to minimise potential bias that may arise from a pre-existing relationship consisting of:- 1. Verbally reminding participants that all information provided during interviews would remain confidential (with caveats e.g., for malpractice) and that there was no obligation to participate (also outlined in all participant information sheets); 2. Verbally emphasising at the beginning of interviews that there are no right or wrong answers and that the aim of the interview was to explore views and experiences from multiple perspectives; and 3. Engaging in reflexivity during monthly supervision with authors (TC, BH and MB) about personal, interpersonal, and contextual biases (Olmos-Vega et al., 2022).

## Findings

Twenty-one occupational therapy practitioners (*n* = 19, occupational therapists; *n* = 2 occupational therapy assistants) participated in this study (Table 2). Twelve were working as an NHS Band 6 specialist occupational therapist, with banding ranging from Band 3 (assistant) to Band 7 (highly specialist occupational therapist). Seven practitioners identified Older Persons Community Mental Health Teams (OP-CMHT) as their primary practice setting, with two reporting that Memory Services (MS) were their primary practice setting. Participants were white British or European and one was male. Mean interview duration was 56 minutes 22 seconds, with a range of 32:44 – 78:27.

**Table 2.** Participant demographic data.

Gender	Age	Ethnicity	Highest Educational Qualification	NHS Band	Primary Practice Setting	% of caseload living with early-stage dementia
Female: *n* = 20	25–29: *n* = 4	White British/European: *n* = 21	Undergraduate Degree/Diploma: *n* = 18	3: *n* = 2	MS: *n* = 2	<20%: *n* = 7
Male: *n* = 1	30–34: *n* = 3		Post Grad Cert/Diploma: *n* = 3	5: *n* = 1	Mental Health Liaison: *n* = 3	20–39%: *n* = 8
	35-39: *n* = 2			6: *n* = 12	OP-CMHT: *n* = 7	40–59%: *n* = 2
	40–44: *n* = 5			7: *n* = 6	Inpatient: *n* = 4	60–79%: *n* = 1
	45–49: *n* = 2				Admissions Prevention: *n* = 2	N/A: *n* = 3^a^
	50–54: *n* = 3				Young-Onset Team: *n* = 1	
	55–59: *n* = 2				Reablement: *n* = 1	
					Specialist Intervention Team: *n* = 1	

Abbreviations: MS: Memory Service; OP-CMHT: Older Persons Community Mental Health Team; OPMH: Older Persons Mental Health.

^a^N/A: Not applicable (used by participants whose caseload at the time of interview did not include people living with early-stage dementia).

### Intervention characteristics

At the time of data generation and where available, no standardised, manualised, or published occupational therapy interventions for early-stage dementia were being delivered by participants. Rather, interventions were based on occupational therapy practitioners' core skills acquired through pre-registration training and practice-based learning. Using a priori themes (Table 1) and inductively developed sub-themes, data about intervention characteristics has been synthesised across sites, with similarities and differences highlighted.

## Rationale

Maintaining or enabling ‘*independence*’, ‘*functioning’* or *‘skills*’ in the context of everyday activities was repeatedly identified as a primary intervention aim. Although not as prominent, additional aims included improving quality of life, wellbeing, and preventing disability associated with increased service use. Some participants highlighted that intervention aims should be viewed in the context of what is important to the person living with early-stage dementia and expressed concerns that interventions in practice, due to a lack of resources, typically focus on personal care or activities associated with risk, for example medication, gas safety or getting lost outside:

‘…the only thing that concerns me in the jobs that I’ve worked has been the focus on personal care is massive and as occupational therapists we’re meant to be holistic and look at their entire life… perhaps their personal care’s all right but the rest of it might be falling apart.’ (P02)

Most participants were unable to identify research that could inform a community-based occupational therapy intervention for early-stage dementia. Rather, participants made broad references to Welsh Government policy, NICE guidelines and described using their own practice or experience-based evidence:

‘…our evidence in this space is quite limited... …a lot of the time it is my own knowledge, own experience that’s done it really…there’s not a lot there… .’ (P13)

Intervention programme theories, or theories of change, were not described by participants, however, occupational therapy conceptual models of practice, namely the Model of Human Occupation (MOHO) (Forsyth, 2021) and the Canadian Model of Occupational Performance and Engagement (CMOP-E) (Baum, 2021), were being utilised. To address a perceived lack of emphasis on memory or cognition by both models, the Cognitive Disabilities Model (CDM) (McCraith & Earhart, 2018) was being utilised at three sites. Broad references were also made to theories relating to errorless learning and procedural or working memory.

### Content and components

#### Pre-intervention “*Groundwork*”

Participants were eager to emphasise that their interventions must be preceded by the development of a therapeutic relationship and an assessment process, culminating in the identification of needs and goals. Talking about their work in MS, one participant described in detail the relational nature of their work which involves an investment of time to develop rapport and build trust to achieve better outcomes:

‘…it is not about numbers and how quick you see someone, there are times when you need to build rapport. It’s times you need to ask them what matters to them… you might have a more pressing issue, but you need to build their trust and work on the thing that matters most to them. …the staff that get the furthest in terms of outcomes... know that it’s about the relationships that they form… and the approach...’ (P14)

Across sites, an assessment process commencing with a locally developed interview to identify needs and intervention goals was typically described. Observational assessments of the home environment and the performance of activities were also viewed as essential:

‘…the home environment can often give you masses of clues or prompts as to what actually is happening. You can go in and …see sometimes if someone is really struggling with certain tasks or things. …You can do much more practical based assessments, you could ask them to operate the washing machine...’ (P06)

Again, these functional assessments were typically locally developed and non-standardised, however, some participants described using additional standardised assessments including the Pool Activity Level (PAL) (Pool, 2012), as well as MOHO and CDM based assessments. The need to use assessments that are also validated outcome measures was raised by some participants, however it appeared none were being used at the time of data generation.

#### Problem-solving strategies

Interventions based on occupational therapy practitioners’ core skills were typically described collectively as ‘*strategies*’ or ‘*memory strategies’*. These strategies were problem-solving in nature and aimed to enable people to overcome or compensate for everyday activity difficulties. A range of strategies were evident, including prompts and reminders and adapting the physical and social environments, which are summarised in the Supplemental Material. Strategies were portrayed as highly individual to the person and their environment, requiring significant personalising and tailoring, often making it difficult for participants to make general statements about their interventions. One participant described the multiple ways she supports medication management and suggested involving the person living with dementia in decisions about strategies:

‘…it’s a good idea to point out or suggest different ways of actually managing medication and for the person to choose the one that they think most suits them…For instance, for some people it’s blister packs, laminated, for some people it might be ticking medication off in a book…for some people it might be actually having a calendar...’ (P05)

Another participant described a process of trial and error to find strategies that work for the person:

‘…it’s about trying what works for that person… very much trial and error. A lot of my job, I think, is having an idea, seeing if it works and then evaluating it and being like, they don’t get on with that, it’s not working, we need to try something else...’ (P15)

#### Emotional support and coping strategies

Whilst not as prevalent, intervention contents to meet needs associated with co-morbid depression, anxiety, and the emotional impact of coming to terms with a dementia diagnosis were described. This consisted of the provision of emotional support, for example through active listening, providing reassurance, and by giving information and advice about the possibility of living well with dementia. It also comprised coping strategies based on Cognitive Behavioural Therapy (CBT) and other modalities:

‘The first thing that you do as an OT, I think, is to listen and you will pick up various cues and triggers and people’s fears, and their concerns and then you need to address those. I like to try and use a CBT approach but… I use quite an eclectic approach, …when somebody first has a diagnosis it is devastating, absolutely devastating, and one of the people… I had to work just two weeks on just talking about fears, emotions, and things like that. …you need to give people information and you need to give them strategies to cope with those thoughts and those feelings, and what strategies… you suggest will completely depend on the individual and what works for them…’ (P05)

#### Advice, signposting and referring

Providing generic advice and information, for example about eligibility for financial assistance, driving, and power of attorney, was also described in the context of intervention contents. Signposting and making referrals to local services that could meet everyday activity needs which were not able to be met by existing occupational therapy services were also discussed. Examples included aids and equipment, community transport and community groups:

“…referring for telecare aids, reducing risks in terms of environment, maybe with smoke alarm detectors or community alarm buttons. We’ve also …referred people onto third sector agencies to keep them more out and about and reducing the risk of isolation.” (P10)

### Mode and location

Interventions were delivered overwhelmingly on an in-person individual or dyad basis in the person’s own home. Participants highlighted that this enabled more individualised interventions by providing the opportunity to work with caregivers who know the person well, to assess activity needs, and to implement interventions in the persons day-to-day environment:

‘…home visits are often really useful because you can see what the home is set up [like], …and who lives there and who has got input with that person... You can obviously look at all the physical adaptations like whether they need a handrail… But also… looking at how that person functions in their day-to-day living, so actually observing people when they’re doing their personal care or their cooking or their cleaning or whatever, so that you can give advice on how things can be improved...’ (P04)

Accessing community venues and locations where everyday activity needs had been identified was also highlighted as important, however it was unclear how frequently this occurred. Only two participants were delivering or were aware of occupational therapy interventions being delivered in a group format, one was being delivered in a clinical setting whilst the other was in both a clinical and non-clinical setting. However, these interventions were not delivered Health Board-wide and were confined to individual therapists’ locality. Perceived benefits of a group format included developing a sense of connection with others in similar circumstances and learning from, and supporting, peers:

“…I think… it’s the same in any mental health setting and physical setting where if a person who’s sat next to you is having a similar problem, it then becomes a shared problem rather than an individual problem. And strategies that a person has lived through and used rather than a piece of paper or a professional saying what the strategies are, become more realistic and more acceptable and achievable.” (P09)

### Duration and intensity

A flexible and individualised approach was advocated when discussing the duration and intensity of individual and/or dyad interventions. Whilst participants at some sites placed a greater emphasis on delivering what they called ‘brief’ interventions of approximately four to eight contacts (to include pre-intervention contents), variation was evident given the personalised nature of interventions:

‘…our interventions are very varied, …I could go and see somebody to do a one-off road safety assessment, it’s done... And yet another person that’s far more complex… I might see them over months. …the longest person I’ve had on my caseload has been a year and that’s because the lady herself, her needs kept changing...’ (P13)

Frequency of contact was typically advocated on a diminishing intensity basis, with potentially multiple visits in the initial weeks, reducing over time. Group interventions, where provided, were being delivered for 5 and 6-weeks’ duration, with contact once a week.

### Interventionist

A consensus was evident about interventionists for individual or dyad interventions. Participants explained that occupational therapists typically conduct assessments, goal setting, initiate intervention programmes and deliver more complex interventions or those associated with risk. Where available, occupational therapy support workers (assistants or technicians) typically delivered interventions that were long-term, requiring practice or a graded approach, for example community re-integration:

‘…assessment work might be done [by me], so quite commonly, same with the risk for getting lost… a gas cooker risk, it may only need two or three visits so I might not involve somebody else in that. But if it’s something which… you want to go in quite often or you want to practice a skill… then the Support Worker did that…’ (P14)

Group interventions were delivered by occupational therapists and support workers together, however, participants at one site spoke about the need to have people living with dementia co-develop and deliver groups to enhance the benefits associated with peer support and learning:

“So, I think any groups or any [group] interventions that are run, the ideal would be to have service user involvement and service user involvement in the delivery…” (P09)

### Contextual barriers

**Figure 1.**
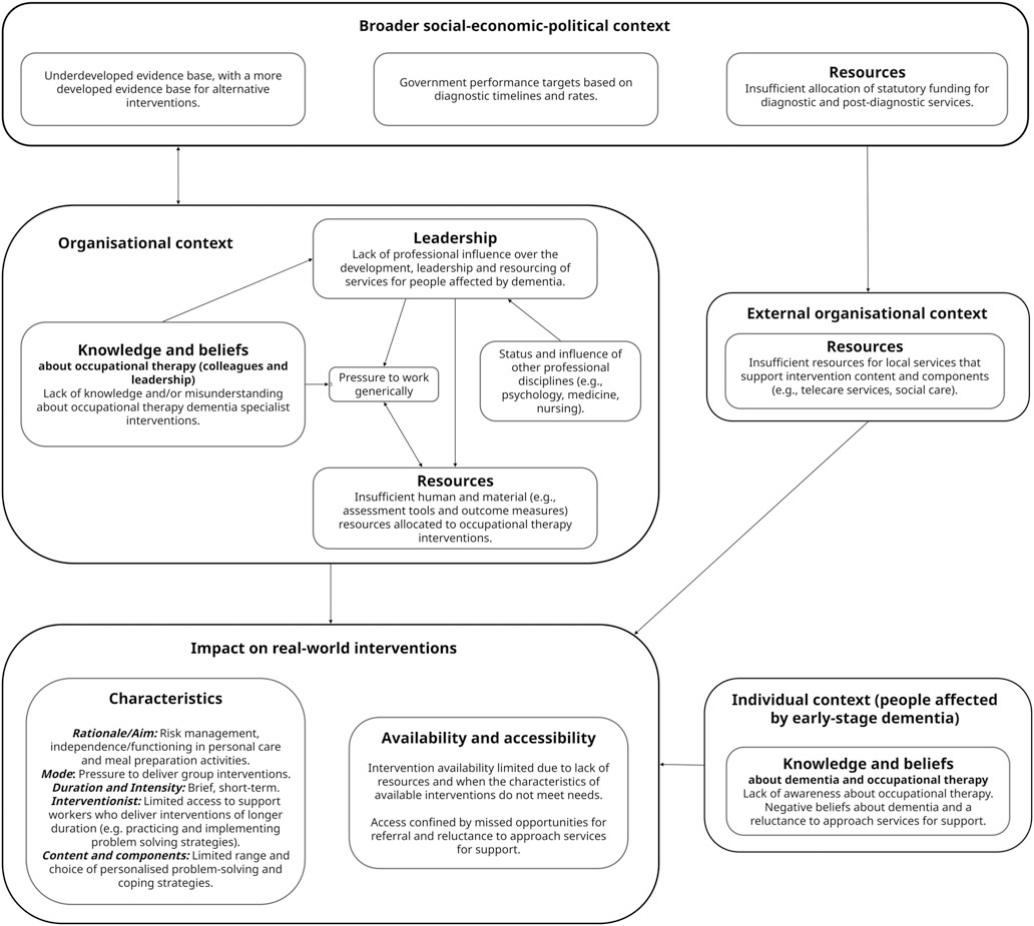
Relationship between identified contextual barriers and real-world occupational therapy intervention characteristics, availability, and accessibility.

Three inter-related themes concerning contextual barriers associated with the delivery or implementation of occupational therapy interventions for people living with early-stage dementia were identified (resources, leadership, and knowledge and beliefs). Figure 1 depicts the relationship between these themes, the contexts in which they were described (e.g., organisational, broader social-political-economic) and their impact on real-world intervention characteristics, availability and accessibility.

### Resources

Confined human resources at an organisational and service level were repeatedly portrayed as significant barriers, and at the time of interviews, two Health Boards did not have occupational therapists working in their MS due to a lack of funding. Participants in these Health Boards commented on the impact this had on their ability to work with people living with early-stage dementia, whom they saw typically in the moderate stages or in crisis in secondary care:

‘…we’re missing people…memory clinic and GPs, we’re not sitting in them and we’re missing people. And then we’re not getting them until they’re further on down the line.’ (P08)

Participants who were working in MS expressed that confined resources were impacting intervention mode, with pressures to deliver interventions in a group rather than individually. Further impacts consisted of limiting who could receive intervention, and its duration, based on risk:

‘…we’re mostly working on risk, so… we’re not able to do long pieces of work with people… just for enabling sake. …in terms of prioritisation we’re listening out and responding and being asked to look at risk to do with occupation… risks to do with functioning at home…’ (P14)

Access to occupational therapy support workers was variable, with the ability to deliver interventions of longer duration dependent upon their availability. Some participants spoke about the lack of external services (e.g., telecare and social care), which restricted the breadth of possible intervention content they could offer, for example being unable to offer telecare or social care to support medication management. A lack of resources at a service level was sometimes attributed to the way in which MS were developed out of existing secondary care services without additional funding following the introduction of the Mental Health (Wales) Measure (2010), and facilitated by the ‘*goodwill*’ of existing staff. In one Health Board, practitioners reflected on barriers arising from the absence of a specific occupational therapy budget at a service level, which restricted their ability to purchase assessment tools and outcome measures:

‘I don’t use any standardised assessment tools because… we don’t have access to much. So whereas we would like to as an OT service move towards using, being affiliated with MOHO and be able to access a lot of that, I think because of, again, we haven’t had an OT budget and the financial implications of that...’ (P13)

### Leadership

The lack of influence participants perceived the profession of occupational therapy had over the leadership and development of services for people living with early-stage dementia was identified as a significant barrier, which they perceived contributed to the lack of resources described. Some participants spoke about the influence psychology as a profession had organisationally in determining the nature of interventions available. For example, in one Health Board the prioritisation of Cognitive Stimulation Therapy (CST) and psychological interventions had resulted in an occupational therapy service ‘*gap’* in MS. Other participants referred to the dominance of the medical model in shaping Welsh Government targets, which they felt had resulted in an over-emphasis on diagnostic assessments and prescribing organisationally, rather than interventions that support people to live well after diagnosis:

‘…the pressure is on Memory Clinics in terms of the timescale …from when they’ve been referred and the point of diagnosis, that’s where the specific [Welsh Government] targets [are], it’s not on what happens next. … It’s a bit like, great you’ve been diagnosed with cancer but there’s no treatment or help afterwards…’ (P14)

An absence of robust evidence that participants could provide to commissioners was identified as a potential explanatory factor concerning the limited influence over service leadership and resource allocation; particularly at a time when there are growing bodies of evidence generated by other professions:

‘…I would say our other biggest challenge is… that lack of research that has been done in that we are getting leapfrogged with certain, like we talked about psychology because we aren’t very good at articulating it, …we haven’t got our evidence to…put to commissioners to say well, this is what we do, this is the outcome of what we do and then be able to get our resources…. I’ve seen that quite a lot over my career… we’ve been leapfrogged over and then we get stuck with bits that perhaps people don’t want to do, yeah the glamorous bits like toilet seats.’ (P13)

At a service level, participants spoke about the challenges of being line-managed by non-occupational therapists and associated pressures to undertake generic as opposed to profession-specific work:

‘I think one of the biggest challenges… is that we’ve been managed by nurses who have been dictating our practice quite a lot, and I’m very fortunate that our Band 7 at the moment is very pro OT… so gets it, understands it and gives me autonomy within my practice but that’s not always the case...’ (P13)

### Knowledge and beliefs (about occupational therapy and dementia)

A lack of knowledge and misunderstandings about dementia specialist occupational therapists by colleagues and senior management, was sometimes associated with an absence of influence over the leadership and development of services and consequently resource allocation. For example, in one Health Board perceptions that occupational therapists provide equipment only was associated with a recent decision to cease occupational therapy intervention in MS since it was perceived that these interventions could be provided by occupational therapists working in general physical health settings and community equipment teams:

‘…refer it to the general OTs, refer it to social services OTs. …that seems to be a barrier, this sort of belief really that it’s everyone’s business which I don’t disagree with, but…is there not a place for a specialist service, so I suppose the barriers that I see is actually the viewpoint of our management, that they don’t seem to understand what we do as OTs...’ (P12)

Another participant reflected on the perception amongst colleagues that occupational therapy is for people living with middle to later stage dementia, leading to missed referrals and opportunities for early intervention:

‘…the subjective opinion of certain professionals may be a barrier because they wouldn’t think that people need to be referred on because, oh they’re OK or they don’t need occupational therapy yet. I think it’s really important that it actually starts at the beginning, and we can always add to...’ (P03)

In addition to members of staff, some participants highlighted that the general public may not be aware of the interventions that occupational therapists provide, and typically do not approach services asking for occupational therapy. Other participants noted that some people may be reluctant to access or engage with services, due to fears of receiving a diagnosis:

‘Some have said, I don’t want to be labelled with dementia, I’m scared, I wouldn’t want that, because they see dementia as, on TV, as like the full blown quite unwell, whereas we know people can live in the community well with dementia for many, many years…’ (P02).

## Discussion

This paper aimed to describe the characteristics of real-world occupational therapy interventions for people living with early-stage dementia in the community, and contextual implementation barriers. Twenty-one occupational therapy practitioners participated across five Health Boards, with results providing insight into the nature of interventions delivered, where available, in routine clinical practice. Multi-level contextual barriers were also identified, which appeared to shape the availability and characteristics of interventions delivered and to whom, highlighting the dynamic relationship between intervention and context (Pfadenhauer et al., 2017; Rogers et al., 2020; Skivington et al., 2021).

### Real-world occupational therapy interventions

At the time of data generation, the availability of occupational therapy intervention for people living with early-stage dementia across Health Boards was variable. Two did not have an occupational therapist in their MS and participants in a third described how their work in MS would be stopping. Whilst occupational therapy interventions appeared to be available in secondary care (e.g., OP-CMHTs), participants working in these services reported working primarily with people in crisis and in the moderate-later stages. This finding suggests that at the time of data generation people living with early-stage dementia were not receiving an equitable service, in contrast to Welsh Government policy (Welsh Government, 2021).

Where interventions were available, broad similarities were apparent in relation to aims (e.g., maintaining or enabling everyday activity ‘*independence’* or ‘*functioning*’), and the need to individually personalise interventions, consistent with occupational therapy professional standards and theory (RCOT, 2021). Participants were also congruent about components, with problem-solving strategies (e.g., prompts and reminders, adaptations to the envrionment), identified as the primary intervention content, largely consistent with the findings of Swinson et al. (2016) and Abendstern et al. (2017). Considered best professional practice for occupational therapists, and akin to COTiD (Wenborn et al., 2021), participants in this study emphasised that occupational therapy interventions must be preceded by an assessment and goal-setting (RCOT, 2021). However, unique to this study, participants were explicit that the development of a therapeutic relationship is also an essential pre-intervention component. Whilst this has not been explicitly described as an intervention component by other intervention programmes for early-stage dementia involving occupational therapists, it has been identified in studies generating qualitative data alongside formal evaluations of such interventions (Burgess et al., 2021; Morgan-Trimmer et al., 2021). Morgan-Trimmer et al. (2021)’s process analysis of cognitive rehabilitation, described how their interventionists, who were primarily highly skilled occupational therapists, went beyond their intervention manual to facilitate a relational approach by dedicating time during each contact for relational work. They describe how this helped participants engage with the intervention, enabled personalisation, and was the vehicle through which social support was provided. This study therefore adds further weight to the inclusion of relational work as a core occupational therapy intervention component.

Despite broad similarities, variation in intervention characteristics were evident, namely in relation to duration, mode, and intervention recipient. Similar to Swinson et al. (2016)’s finding of a median duration of 2.5 hours, yet in contrast to COTiD’s 10 hours’ duration (Wenborn et al., 2021), some very brief contacts to meet specific needs e.g., road safety, were reported. Another departure from COTiD, which is delivered in a dyad format only, consisted of interventions delivered on a group and individual basis. Heterogeneity was also described in relation to intervention aims and therefore who received interventions, for example some participants worked only with people who had everyday activity needs associated with risk or personal care, in contrast to COTiD. These findings suggest that real-world community-based occupational therapy interventions are different to interventions that have been evaluated in UK based clinical trials, indicating the need to evaluate real-world interventions to establish their evidence base.

### Contextual barriers

Whilst some variation is expected of a personalised intervention (e.g., individual preferences for mode, and needs that do not justify a lengthy intervention), multi-level contextual barriers identified provide some insight into the possible reasons why heterogeneity in availability and characteristics were reported. Akin to Rogers et al., (2020), contextual barriers were identified at all system levels (individual, service, organisational and external), with some barriers (e.g., resources) permeating multiple levels. A lack of resources, namely human appeared to be the most significant challenge described across Health Boards, however, the impact this had differed. In some Health Boards, occupational therapy intervention for early-stage dementia in primary care (e.g., primary care) was simply not available, whilst in others mode, duration, and recipient appeared to be shaped by resource limitations. This highlights that different organisations (systems) and services within these organisations can respond in differing ways to a shared problem (lack of resources). The scarcity of resources and its impact on intervention delivery identified is consistent with the findings of Cummins and Warren (2010) in Ireland, adding to knowledge generated about the impact context has on occupational therapists’ ability to practice in accordance with their professional training and values.

Unique to this study, however, is the insight generated into why community-based occupational therapy interventions for early-stage dementia may be particularly affected by this contextual challenge. Potential inter-related mechanisms were identified consisting of: 1. A lack of influence compared to other professional disciplines (e.g., psychology and medicine) over the leadership of teams, organisations, national policy, and therefore the allocation of resources at these system levels; 2. Misunderstandings and/or a lack of knowledge about dementia specialist occupational therapists and the interventions they provide; and 3. An absence of research to justify the allocation of resources. Whilst these are new findings in the context of occupational therapy interventions for early-stage dementia, the lack of presence in leadership roles (Tempest & Dancza, 2019), limited understanding about occupational therapy (Darawsheh, 2018; Patel & Shriber, 2001; Pottebaum & Svinarich, 2005; Tariah et al., 2012) and an under-developed evidence-base (Watson, 2021) have all been previously acknowledged as long-standing challenges for the profession of occupational therapy.

### Limitations

Study limitations must be acknowledged, including the inclusion of occupational therapy practitioners only as participants. Future research should seek to capture perspectives about contextual barriers as experienced by people living with early-stage dementia themselves, as well as their supporters, particularly since existing research conducted in the context of controlled trials has indicated that barriers can be associated with the interventionist (Voigt-Radloff et al., 2011). Interview questions about contextual barriers were open ended, allowing participants themselves to define what they perceived to be a contextual barrier. The use of a published framework, for example by Pfadenhauer et al., (2017) and Rogers et al., (2020), to guide interview questions may have yielded additional themes, including those at an individual interventionist level.

### Implications for future research

This study was conducted to inform the development of a programme theory to underpin a future evaluation of real-world occupational therapy interventions for people living with early-stage dementia in the UK. Firstly, it has indicated that real-world interventions have some different characteristics (mode, duration, aim) to COTiD, the only intervention programme evaluated using a RCT in a UK context to date (Wenborn et al., 2021). Consequently, outcomes reported by Wenborn et al. (2021) cannot be used as a reliable proxy for interventions delivered in routine clinical practice, indicating that an evaluation of real-world interventions is needed. In order to conduct such an evaluation, this study has generated detailed evidence about intervention characteristics that can be utilised to inform a programme theory to underpin such an evaluation as per current international guidelines (Skivington et al., 2021).

Secondly, it has identified that real-world interventions were being provided in the majority of participating Health Boards' MS, suggesting that identifying an appropriate TAU group for a RCT without the confounding influence of routine occupational therapy interventions may be challenging, as experienced by Wenborn et al. (2021). Recognising that it may not be possible to conduct RCTs in certain instances, the National Institute for Health and Care Excellence (2022) have published their real-world evidence framework. Real-world evidence consists of data generated in relation to routine interventions and procedures, which can be utilised using a range of study designs and methodologies, for example an observational cohort study or a realist evaluation (NICE, 2022). The latter design may be the most appropriate to progress this field of research given the contextual barriers identified, since it facilitates an evaluation that seeks to understand the interaction between context, intervention and outcome (Pawson & Tilley, 1997). Indeed, contextual barriers identified, reflect long-standing professional and possibly systemic challenges encountered by occupational therapists working in a minority profession in the UK health system. A realist informed evaluation would enable the generation of information about the impact these contextual barriers have on intervention characteristics and outcomes, and about how practitioners locally overcome or adapt to such challenges.

## Supplemental Material

Supplemental Material - Real-world occupational therapy interventions for early-stage dementia: Characteristics and contextual barriers Supplemental Material for Real-world occupational therapy interventions for early-stage dementia: Characteristics and contextual barriers in Bethan M Edwards, Monica Busse, Teena J Clouston and Ben Hannigan in Dementia
