# Supplementary material for: Real-world occupational therapy interventions for early-stage dementia: Characteristics and contextual barriers
Source: Dementia (London). 2024 Aug 20;24(3):388–407. doi: 10.1177/14713012241272815 (PMC11915762; doi:10.1177/14713012241272815)
Supplement: Supplemental Material - Real-world occupational therapy interventions for early-stage dementia: Characteristics and contextual barriers [file sj-pdf-1-dem-10.1177_14713012241272815.pdf]

Supplementary Material

Table 1: Problem-solving strategy type, function and supporting quotations.

Strategy Type	***Function and example(s)***	***Supporting quotation(s)***
Prompts and Reminders	Prompting and supporting activity performance by compensating for cognitive difficulties (memory, organisation, orientation). E.g., diaries, calendars, white boards, checklists, step by step instructions and labels. Aids and equipment, for example timers, smart technology, and alarms were also described as prompts or reminders.	*'…a carousel is a piece of equipment that's like… a round plastic thing, that basically, you put the medication in and at the time at which the medication is due it goes around, the flap opens, and it just does this incredibly loud buzz…And it won't stop buzzing until that person takes their medication….' (P15)*
Adapting the Physical Environment	1. Minimising distractions and complexities in the physical environment. E.g., turning off a TV or telephone, decluttering.<br><br>2. Prompting and supporting activity performance by compensating for cognitive difficulties (memory, organisation, orientation). E.g., placing clothes	*'…in the early stages you might be able to give more visual clues, so maybe if you declutter the kitchen they would still be able to actually make that cup of tea because they can see where everything is and they have learned that behaviour, …they've done it so many times before, …with the less clutter around they're able to concentrate on the task.' (P04)*<br>*'It can also be aids and equipment, because they're older*

Strategy Type	*Function and example(s)*	*Supporting quotation(s)*
	somewhere visible rather than in a cupboard and changing cupboard doors from solid wood to glass.<br><br>3. Minimising demands in the physical environment arising from physical or sensory needs associated with dementia as well as the consequences of aging. E.g., handrails, equipment.	*people, so just because, the dementia might not be the factor that's preventing somebody from completing their personal care it could be just that they can't get to the bathroom or on and off the loo or whatever. So we do a fair bit of equipment provision as well as all those other things.' (P18)*
Adapting the Social Environment	Minimising demands in the social environment and maximising opportunities for activity performance. E.g., increasing family and caregivers' knowledge and understanding about dementia, its impact on activities and problem-solving and coping strategies through the provision of information, advice, training, and education.	*'…it's about educating the family members to try to stop them from deskilling people by stopping them doing things because they're making a mistake. I remember going to a …lady… whose husband wouldn't let her wash the dishes anymore because she didn't wash them clean. …he stopped her doing so much that generally she sat in front of the television all day and did nothing'. (P03)*<br><br>*'…what I find…is that families find it really difficult to understand that the person [living with dementia] seems to do one thing but not another, one day they did this, then they couldn't do that, or they'd get frustrated when they've asked them something and they haven't done that. So sometimes what we try to do is… illustrate to families how*

Strategy Type	*Function and example(s)*	*Supporting quotation(s)*
		the dementia may be affecting their functioning, and what you can expect of someone, and what way a carer can help that person to function. …we can work out that they score 64 on an ACE but what does that mean? It's the every-day practicalities of this illness that we try to deal with...' (P12)
Adapting the activity environment	Minimising the complexity and demands of an activity. E.g., removing the number of actions required to achieve the desired outcome.	*'…she [person living with dementia] was relying a lot on family to prepare food for herself and one of her goals was she wanted to get back to be able to managing that more herself. …she's struggling a lot more with using her oven and the hob, but we're looking at her getting her using the microwave, so that she can make…microwave meals. So obviously did some kitchen assessments to see how, her baseline, where she is and then…got some memory prompts to step by step guide to help her process exactly what she needs to do. Lots of practising, using the microwave, and then putting stickers in places to help her know where the dial is and yeah, and she's doing really well'. (P15)*
Habituation:	Providing structure and familiarity through spatial-	*'…some of my patients have always gone to get their*

Strategy Type	*Function and example(s)*	*Supporting quotation(s)*
"Routines"	temporal patterns of activity. E.g., keeping the remote control in the same place, eating at the same time, doing things in the same way.	*pensions on a Monday, now if that's the case you… keep that going. I've recently had a client whose daughter started collecting her pension and that confused the lady so much and it just caused so much distress the daughters now backed off and allows the client to go and collect her pension, go to the corner shop which was always her routine and then come back home with her purchases.' (P05)*
